# In-shoe plantar pressure measurement technologies for the diabetic foot: A systematic review

**DOI:** 10.1016/j.heliyon.2024.e29672

**Published:** 2024-04-15

**Authors:** Pedro Castro-Martins, Arcelina Marques, Luís Coelho, Mário Vaz, João Santos Baptista

**Affiliations:** a CIETI, ISEP, Polytechnic of Porto, Portugal; b Faculty of Engineering, University of Porto, Portugal; c Institute for Science and Innovation in Mechanical and Industrial Engineering, Porto, Portugal; d INESC-TEC, Centre for Robotics in Industry and Intelligent Systems, Porto, Portugal

**Keywords:** Diabetic foot, Plantar pressure, In-shoe systems, Instrumented insoles, Systematic review

## Abstract

**Introduction:**

Loss of cutaneous protective sensation and high plantar pressures increase the risk for diabetic foot patients. Trauma and ulceration are imminent threats, making assessment and monitoring essential. This systematic review aims to identify systems and technologies for measuring in-shoe plantar pressures, focusing on the at-risk diabetic foot population.

**Methods:**

A systematic search was conducted across four electronic databases (Scopus, Web of Science, PubMed, Oxford Journals) using PRISMA methodology, covering articles published in English from 1979 to 2024. Only studies addressing systems or sensors exclusively measuring plantar pressures inside the shoe were included.

**Results:**

A total of 87 studies using commercially available devices and 45 articles proposing new systems or sensors were reviewed. The prevailing market offerings consist mainly of instrumented insoles. Emerging technologies under development often feature configurations with four, six or eight resistive sensors strategically placed within removable insoles. Despite some variability due to the inherent heterogeneity of human gait, these devices assess plantar pressure, although they present significant differences between them in measurement results. Individuals with diabetic foot conditions appears exhibit elevated plantar pressures, with reported peak pressures reaching approximately 1000 kPa. The results also showed significant differences between the diabetic and non-diabetic groups.

**Conclusion:**

Instrumented insoles, particularly those incorporating resistive sensor technology, dominate the field. Systems employing eight sensors at critical locations represent a pragmatic approach, although market options extend to systems with up to 960 sensors. Differences between devices can be a critical factor in measurement and highlights the importance of individualized patient assessment using consistent measurement devices.

## 1 Introduction

Loss of cutaneous sensation and subsequent ulceration of some regions in the feet of people with diabetes are common consequences of the disease, attributed to complications such as peripheral neuropathy, peripheral vascular disease, decreased joint mobility and high pressure injuries [[1], [2], [3], [4], [5], [6], [7]]. Diabetic neuropathy disrupts the patient's protective cutaneous feedback mechanism, significantly reducing sensitivity to touch and pain [8]. As a result of these complications, it is estimated that approximately 34% of people with diabetes will develop a foot pressure ulcer during their lifetime [9]. These lesions are highly prone to infection, and in severe cases, may necessitate amputation, making diabetic foot ulcers a leading cause of nontraumatic lower limb amputation [10].

Following the decrease or loss of protective skin sensitivity, the integrity of the skin and the patient's perception of painful stimuli are compromised, rendering them susceptible to traumatic ulceration, a complication commonly termed diabetic foot [[11], [12], [13], [14]]. These diabetic foot ulcers predominantly occur in the plantar region of the forefoot [15,16], corresponding to regions where high pressures typically occur between the foot and the patient's footwear during gait [17]. However, the midfoot and plantar rearfoot regions are also affected [18,19] and, less frequently, the dorsal region [17]. Consequently, mapping high plantar pressures zones is employed to guide the manufacture of footwear and insoles adapted to offload pressure in these critical regions [[20], [21], [22]].

Supervision of patients and reduction of plantar pressures are essential actions to accelerate ulcer healing and prevent recurrence [23,24]. Currently, plantar pressure monitoring through footwear-integrated technologies is beginning to replace the traditional procedure of measuring platforms, which are typically only used in clinical and barefoot settings. Examples include sensors integrated into the body of footwear, insoles, or socks [[25], [26], [27], [28]].

To better understand and address plantar pressures within footwear in individuals with diabetic foot, it is essential to explore available measuring devices, the results they yield, and their application conditions. In this context, this review seeks to employ systematic methods to identify all the measuring devices used in these conditions, systematically highlight and compare the characteristics of their technologies and application methodologies, and additionally, establish a synthesis of current technologies and those in the research and development phase serving this purpose. The objective is to provide a comprehensive and comparative overview of various plantar pressure measurement technologies used inside shoes, presenting their characteristics and some associated plantar pressure measurement results.

## 2 Methods

This systematic review was conducted and structured according to the guidelines proposed by the PRISMA methodology – Preferred Reporting Items for Systematic Reviews and Meta-Analysis [29]. A meta-analysis is not included in this systematic review.

### 2.1 Research strategy

This work focuses exclusively on in-shoe devices intended for diabetic foot conditions from both clinical and occupational health and safety perspectives. To develop an objective research question, the PICO concept was employed: Population, in-shoe plantar pressure devices; Interest, technologies used to assess in-shoe diabetic foot plantar pressure; Comparison, variability in technology characteristics; Outcomes, results offered by the technologies. This approach led to the following research question: In the context of existing in-shoe plantar pressure measuring devices (P), what are the technologies and methodologies used for measuring diabetic foot plantar pressure (I), how do the distinct characteristics of these devices compare (C) and what are the measurement results (O) obtained? This research question aims to provide a comprehensive overview and comparison of various plantar pressure measuring devices, their technologies, features and the results they offer, thereby informing researchers, doctors and others healthcare professionals about the available options.

**Fig. 1:**
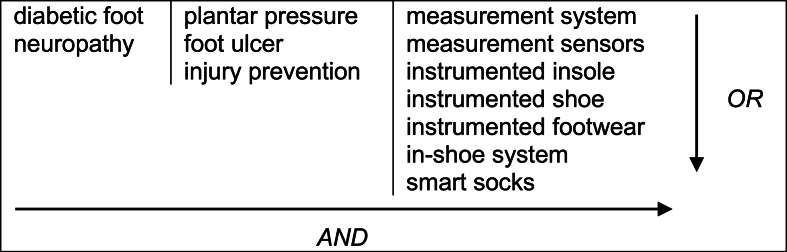
Keywords used in the search for the selection of articles in the databases.

In December 2022 (updated in February 2024), four electronic databases (Scopus, Web of Science, PubMed, Oxford Journals) were screened using selected keywords (see Fig. 1) to capture relevant literature on the topic. The search query used, with the combination of keywords to search all databases, was: (“diabetic foot” OR “neuropathy”) AND (“plantar pressure” OR “foot ulcer” OR “injury prevention”) AND (“measurement system” OR “measurement sensors” OR “instrumented insole” OR “instrumented shoe” OR “instrumented footwear” OR “in-shoe system” OR “smart socks”). The records obtained through the database search were managed using Mendeley software. However, the article selection process was conducted manually, with Mendeley assisting only in identifying duplicate records, which were subsequently validated and removed based on the authors' decision.

### 2.2 Selection and eligibility criteria

The main information included in this systematic review was derived from articles (document type) published in journals (source type). The review covered articles published in the English language without date restrictions. The initial review process consisted of two main steps: abstract-level screening and full-text review. During the abstract-level screening, the titles and abstracts of the articles were analyzed to classify them as on-topic or not, based on the proposed eligibility criteria outlined below. Articles that clearly did not fit the topic were rejected at this stage. In the subsequent step, the full text of the remaining articles was thoroughly reviewed and eligibility criteria were applied. Articles were selected if they met the following cumulative requirements: i) utilization of systems for plantar pressure measurement or development of new systems and/or sensors; ii) inclusion off systems and/or sensors incorporated into the footwear and/or removable devices insertable into footwear; iii) with applicability in the context of diabetic foot pathology for monitoring plantar pressures. The article selection process was conducted independently by two authors, PCM and AM. Any discrepancies regarding the inclusion of a particular article were resolved by a third author, LC, to ensure objectivity in the article selection process.

The articles included considered studies that proposed new sensors or systems still under research or development, which served as the main source of information. Additionally, articles utilizing measurement systems already available on the market were included to identify the standard system and the usage rates. Studies that did not use systems or sensors attached to the inside of the shoe or that could be inserted, such as insoles or other removable methods, to measure plantar pressures were excluded. Similarly, articles reporting studies of plantar pressure assessment conducted barefoot, i.e., without any footwear, were also excluded. Furthermore, the reference lists of eligible articles cited by the authors were analyzed to identify further relevant information, which was subject to the same eligibility criteria. This analysis was conducted only once and the same selection method mentioned earlier was followed, with any discrepancies resolved by a third author.

The scope of this review covered information pertaining to devices specifically designed to measure in-shoe high plantar pressures, distinct from other plantar pressure measurement systems used barefoot in clinical setting. We considered this approach more appropriate as it mirrors the daily life experience of individuals with diabetes, whether diagnosed with diabetic foot or at high risk of developing it, as they typically wear shoes, including shoes customized to reduce plantar pressure.

### 2.3 Data extraction

Once the selection process of eligible articles was completed, the following data were collected: i) identification of the authors, year of publication and title; ii) research subject and objectives; iii) methodology applied, including the equipment used or type of device developed and/or proposed sensors, object of measurement, technology used, number of sensors and their locations, operating conditions and target and control sample (if applicable); iv) results obtained and conclusions. These extracted data were essential in addressing a set of predefined questions outlined in the results section and facilitated an objective synthesis of the most relevant information. Additionally, data was aggregated concerning the measurement of plantar pressure in individuals with diabetes and healthy. This involved synthesizing the results of studies presenting data on average plantar pressure in critical areas such as the hallux toe, metatarsal heads, lateral midfoot and heel, as reported in the analyzed studies. It is important to note that no additional processing was conducted on these data, only a Mann-Whitney test was performed to assess whether differences are statistically significant in plantar pressure patterns in different groups of individuals, including those with diabetes and healthy individuals, with measurements performed by some of the devices and technologies identified in this review. For the Mann-Whitney test, a confidence level of 99% was applied, corresponding to a significance level (*α*) of 0.01. Results with *P* value < 0.01 were considered to indicate a statistically significant difference between the comparison groups.

## 3 Results

**Fig. 2:**
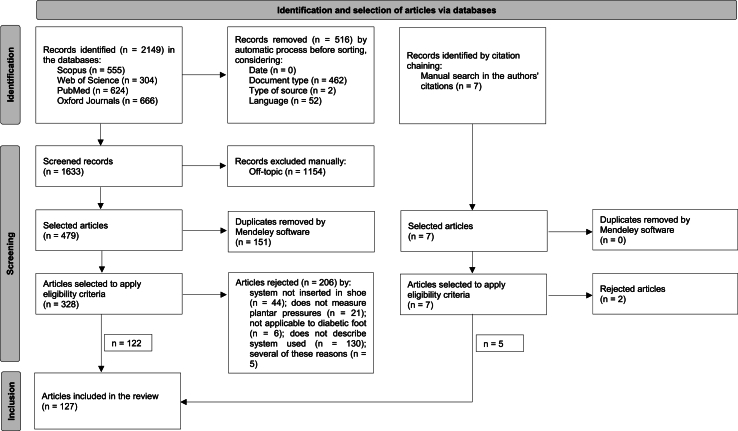
Flow diagram of the selection of articles according to the PRISMA methodology.

The search and selection process, depicted in Fig. 2, initially identified a total of 2149 articles, spanning from 1979 to February 2024. From this process, eligible articles (n = 127) were categorized into two groups: i) 87 studies using commercially available plantar pressure measurement systems; and ii) 45 research articles focused on the development of new systems and/or sensors for measuring plantar pressures inside the shoe or capable of attachment. It's noteworthy that some authors addressed the use of multiple models, including hybrid models combining existing systems available on the market with new sensors under development. Consequently, these articles were counted in both groups.

For the first group of 87 articles, only an informative summary is provided to identify each system used. In this group, authors do not detailed descriptions of the characteristics and utility of the devices employed. Instead, their focus lies solely on the variables measured in each group of volunteer patients. The analysis only consisted of identifying the device used, its applicability, the respective utilization rate and the values obtained in plantar pressure measurements. The studies belonging to the second group of 45 articles were included in this systematic review for a more detailed analysis. These articles present the most recent advances in technology for monitoring plantar pressures inside footwear suitable for the diabetic foot conditions. The information collected from both groups provided important insights into the current state of technology and its evolutionary trend. A summary of the information collected characterizing the first and second groups is presented in Table 1, Table 2, respectively.

**Table 1:** Summary of the characteristics of the devices available on the market to measure and evaluate plantar pressures inside the shoe. (a) considering the number of publications resulting from research whose authors use these devices in their studies.

Device and manufacturer	Number of publications	Publication references	Utilization rate ^(a)^	Device features							
				Application type	Number of pressure sensors	Technology	Other sensors	Battery life	Communication	Acquisition rate	Usage cycles
F-Scan TekScan, USA [31]	42	[35,36,47,48,72,86,87,94],[[96], [97], [98], [99], [100], [101], [102], [103], [104], [105], [106], [107], [108], [109], [110], [111], [112], [113], [114], [115], [116], [117], [118], [119], [120], [121], [122], [123], [124], [125], [126], [127], [128], [129]]	44.7%	Insole	960 (variable with insole number)	Resistive	–	2 h	USB	100 Hz	5 to 15 (with intense activity)
Pedar insole novel GmbH, Germany [32]	35	[96,97,[130], [131], [132], [133], [134], [135], [136], [137], [138], [139], [140], [141], [142], [143], [144], [145], [146], [147], [148], [149], [150], [151], [152], [153], [154], [155], [156], [157], [158], [159], [160], [161], [162]]	37.2%	Insole	99	Piezoelectric or capacitive	–	4.5 h	USB, Bluetooth, Fiber optic	50 or 100 Hz	–
Biofoot Biofoot IBV, Spain [163]	1	[85]	1.1%	Insole	64	Piezoelectric	–	–	USB, Wi-Fi	750 Hz	–
RS Scan Rs Scan, Belgium [164]	1	[165]	1.1%	Insole	356 or 900	Capacitive	–	–	–	–	–
SurroSense RX or Sensory Insole Orpyx Medical Technologies [166]	6	[130,[167], [168], [169], [170], [171]]	6.4%	Insole	8 or 37	–	Temperature	8–12 h	Bluetooth	100 Hz	–
Sensoria Socks Sensoria Inc., USA [91]	2	[73,170]	2.1%	Sock	3	Piezoresistive textile	–	–	Bluetooth	–	–
paroTec Paromed, Australia [172]	3	[110,173,174]	3.2%	Insole	24	Piezoresistive	–	–	SD memory card	300 Hz	–
Medilogic Medilogic GmbH, Germany [90]	1	[139]	1.1%	Insole	240	Resistive	–	16 h	Wireless (no detail)	100 or 300 Hz	–
Walkasins Rx Function, USA [175]	1	[176]	1.1%	Orthosis/Insole	–	–	–	–	Wireless (no detail)	–	–
WalkinSense Tomorrow Options Microelectronics [177]	2	[177,178]	2.1%	Insole	8	Piezoresistive	–	–	Wireless (no detail)	–	–

**Table 2:** Summary of the characteristics of devices under development to measure and evaluate plantar pressures inside the shoe.

Author, year	Device (typology)	Measurement object	Technology	Number of sensors	Sensor locations	Operational conditions						
						Communication	Battery life or capacity	Acquisition rate	Measuring range	Sensitivity and Resolution	Temperature and Humidity	Usage cycles
Amemiya et al., 2016, 2020 [46,81]	Plantar system	Plantar pressure and shear force	Triaxial piezoelectric sensor (Shokac Chip, Touchence, Japan)	6	Two sensors in each location: 1st, 2nd and 5th metatarsal head	Bluetooth (with control unit attached to the user's leg)	Yes (no details)	100 Hz	Tests with results of normal pressure at 510 kPa and shear at 127 kPa	–	–	–
Anas, 2014 [42]	Insole	Plantar pressure/force and dorsiflexion	Resistive force sensor (FSR 402, Interlink electronics) + flexible resistive strain gauge (FBS FLX-01, Spectra Symbol)	4 + 1	FLX sensor: longitudinal of the sole of the foot; FSR sensor: hallux, 1st and 5th metatarsal heads, and heel	Wired	Yes (no details)	10 Hz	Author presents measurements with patients up to 80 N force and dorsiflexion angle up to 50°	–	–	–
Aqueveque et al., 2018 [41]	Insole	Plantar pressure	Capacitive sensor	8	Hallux, between the 4th and 5th toes, three on the metatarsal heads, lateral and medial midfoot and heel	Bluetooth	500 mAh	100 Hz	–	–	–	–
Atlas et al., 2008 [56]	Plantar system	Plantar pressure	Resistive force sensor (Flexiforce, Tekscan)	–	Heel	Wired (control unit on the user's belt)	–	–	–	–	–	–
Brown et al., 2004 [58]	Plantar system (using specific diabetic footwear soles)	Plantar pressure	Resistive force sensor (FSR, Interlink electronics)	7	Hallux, heel, base of the 5th metatarsal, heads of the 1st, 2nd, 3rd/4th and 5th metatarsals	Wired	–	60 Hz	–	–	–	–
Dabiri et al., 2008 [57]	Plantar system	Plantar pressure	Resistive force sensor (Flexiforce, Tekscan)	5	1st, 3rd and 5th metatarsal heads, lateral midfoot and heel	Zigbee or Bluetooth	Yes (no details)	–	–	–	–	–
Darwich et al., 2023 [61]	Insole	Plantar pressure	Resistive force sensor (FSR 402, Interlink Electronics)	7	Hallux, 1st and 5th metatarsal heads, medial and lateral midfoot and two on the heel	Wireless (no details)	–	200 Hz	–	–	–	–
Du et al., 2015 [51]	Sensor	Plantar pressure and shear force	Triaxial inductive sensor	Not applicable	Not applicable	Not applicable	Not applicable	Not applicable	Normal force: 0–800 N; Shear: 0–130 N	1 N	–	–
Femery et al., 2004 [55]	Plantar system for attaching to the shoe	Plantar pressure	Resistive force sensor (FSR, Interlink Electronics)	6	Hallux, 1st, 3rd and 5th metatarsal heads and medial and lateral heel	Wired	–	100 Hz	0–1.2 MPa	0.1–10 kg cm^−2^ ±0.5% of total applied force	−30 to 170 °C	–
Gerlach et al., 2015 [82]	Insole	Plantar pressure	Piezoresistive sensor	6	Hallux, three on the metatarsal heads, lateral midfoot and heel	Wired	–	100 Hz	–	–	–	–
Ghazi et al., 2024 [62]	Insole	Plantar pressure	Resistive force sensor (FSR, Flexiforce)	5	1st and 5th metatarsal heads and three on the heel	Wi-Fi, with smartphone app	AAA battery (2 un.)	0.2 Hz	–	–	–	–
Guignier et al., 2019 [71]	sock	Plantar pressure	Fiber optic sensor (POF, Geniomer)	3	Metatarsus, midfoot and heel	Wired	–	–	Tests between 3 and 120 kPa	–	–	–
Hamatani et al., 2016 [36]	Hybrid insole (integration of F-Scan insole and uniaxial and biaxial shear sensors)	Plantar pressure and shear force	F-Scan: resistive; Uniaxial and biaxial shear sensor: no details	F-Scan: 954 (variable with insole number); Uniaxial: 3; Biaxial: 1	F-Scan: the entire plantar region; Uniaxial: 1st, 2nd and 5th metatarsal heads; Biaxial: heel	Wired	–	50 Hz	F-Scan: up to 862 kPa; Sensors: no details	F-Scan: 3.9 sensors per cm^2^ (spatial resolution) Sensors: no details	–	F-Scan: 5 to 15; Sensors: no details
Hu et al., 2024 [64]	Insole	Plantar pressure	Resistive force sensor (based on Velostat polymer film; Adafruit Industries)	174	The entire plantar region	Bluetooth	3.7 V, 230 mAh; up to 3 h	50 Hz	–	–	–	–
Klimiec et al., 2014 [39]	Insole	Plantar pressure	Piezoelectric sensor (PVDF)	8	Hallux, 4th toe, 1st, 3rd and 5th metatarsal heads, lateral and medial midfoot and heel	Wi-Fi or Bluetooth	720 mAh	–	–	–	Up to 85 °C	–
Laaraibi et al., 2023 [63]	Insole	Plantar pressure	Resistive force sensor (based on Velostat polymer film; 3 M's Electronics)	6	Hallux, 1st and 5th metatarsal heads and two on the heel	–	–	–	–	–	–	–
Leal-junior et al., 2018 [69]	Insole	Plantar pressure	Fiber optic sensor (POF)	4	Medial and lateral forefoot, lateral midfoot and heel	Wired (to control unit fixed on the back of the shoe)	9 V	–	–	0.009 N^−1^	–	–
Li et al., 2023 [65]	Insole	Plantar pressure	Resistive force sensor (based on conductive carbon film)	104	The entire plantar region	Wi-Fi, with computer	Yes (no details); up to 3 h	28 Hz	0–1600 kPa	0.01 kPa^−1^ in the range 500–1600 kPa	−15 to 40 °C	3000 times at ≈ 850 kPa
Liang et al., 2016 [67]	Plantar system	Plantar pressure	Fiber optic sensor (FBG)	6	1st, 2nd, and 3rd metatarsal heads, lateral and medial midfoot and heel	Wired	–	–	–	–	No temperature influence	–
Lin et al., 2017 [75]	sock	Plantar pressure	Piezoresistive textile sensor	4	1st, 3rd and 5th metatarsal head and heel	RF 866 MHz	Battery-free (RF energy harvested, with RFID)	–	Up to 1 MPa	Up to 400 kPa, 10 kPa resolution; 400 kPa–1000 kPa, 60 kPa resolution	–	–
Lord et al., 2000 [47]	Hybrid footwear-coupled plantar system (combination of F-Scan insole and biaxial shear sensors)	Plantar pressure and shear force	F-Scan: resistive; Biaxial shear sensor: magneto-resistive	F-Scan: 954 (variable with insole number); Biaxial: 3	F-Scan: the entire plantar region; Biaxial: heel, 1st (or 2nd) and 3rd (or 4th) metatarsal head	Wired	–	F-Scan: 100 Hz; Sensors: 400 Hz	F-Scan: up to 862 kPa; Sensors: no details	F-Scan: 3.9 sensors per cm^2^ (spatial resolution) Sensors: no details	–	F-Scan: 5 a 15; Sensors: no details
Luna-Perejón et al., 2023 [66]	Insole	Plantar pressure	Capacitive sensor (with PMDS dielectric)	12	Seven in the forefoot, three in the midfoot area and two in the heel	Bluetooth	Two LiPo battery (3.7 V, 450 mAh)	10–25 Hz	–	0.46–0.76 pF kPa^−1^	–	–
Mahmud et al., 2023 [72]	Insole (two models, I and II)	Plantar pressure and temperature	Model I: fiber optic (FBG); Model II: resistive force sensor (FSR) and negative temperature coefficient thermistors (NTC)	Model I: 15 sensing units for pressure and 5 for temperature; Model II: 16 FSR and 8 NTC	Model I: at approx. the same location as model II; Model II: nine FSR in the forefoot, four in the midfoot area and three in the heel; five NTC in the forefoot, two in the midfoot area and one in the heel	Model I: wired with USB serial; Model II: Bluetooth	Model I: not applicable; Model II: no details	Model I: 40–100 Hz; Model II: 40 Hz	Model I: up to ≈64.6 kg and ≈252.2 °C; Model II: ≈690–1380 kPa and −30 to 90 °C	Model I: resolution of 0.1 °C and 1 με for strain; Model II: ≈ resolution of 0.5% of the applied pressure	–	–
Mori et al., 2012 [35]	Hybrid insole (integration of F-Scan insole and uniaxial and biaxial shear sensors)	Plantar pressure and shear force	F-Scan: resistive; Uniaxial and biaxial shear sensor: magnetic variation (Keisoku Support Ltd., Hiroshima)	F-Scan: 960 (variable with insole number); Uniaxial: 2; Biaxial: 1	F-Scan: the entire plantar region; Uniaxial: 2nd and 5th metatarsal heads; Biaxial: heel	Wired	–	F-Scan: 50 Hz; Sensors: 500 Hz	F-Scan: up to 862 kPa; Sensors: uniaxial up to 10 kgf and biaxial up to 8 kgf	F-Scan: 3.9 sensors per cm^2^ (spatial resolution) Sensors: 0.1 kgf	–	F-Scan: 5 a 15; Sensors: no details
Morley et al., 2001 [93]	Multisensor system	Plantar pressure, temperature and humidity	Pressure sensor: resistive (Paromed); Temperature sensor: Resistive RTD (Paromed); Humidity sensor: no details (Honeywell HIH series)	4 + 2 + 1	Pressure: heel and three metatarsal heads; Temperature: under the 3rd metatarsal head and on the heel; Humidity: toe cap of the insole	Wired	Yes (no details)	Pressure: 30 Hz; Temp. and humidity: 1 reading per min.	–	–	Stability up to 35 °C and up to 95% humidity	Some days with 8 h of daily use
Ostadabas et al., 2012 [44]	Insole	Plantar pressure	Resistive force sensor (Flexiforce, Tekscan)	5	Hallux, heel and 1st, 2nd and 3rd/5th metatarsal heads	Wired	–	250 Hz	–	–	–	–
Pataky et al., 2000 [54]	Plantar system for attaching to the shoe	Plantar pressure	Resistive force sensor (FSR 174, International Electronics)	4	1st, 3rd and 5th metatarsal head and heel	Wired, up to control unit on user's belt	4 LR6 1.5 V batteries; up to 8 days or 60,000 gait cycles	96 Hz	–	–	–	≈ 10^7^
Pataky et al., 2003, 2005 [59,60]	Plantar system	Plantar pressure	Resistive force sensor (FSR 174, International Electronics)	5	Hallux, on the 1st, 3rd and 5th metatarsal heads and heel	Wired	–	96 Hz	–	–	–	–
Perrier et al., 2014 [74]	sock	Plantar pressure	Piezoresistive knitted textile	8	Hallux, four on the metatarsal heads, lateral midfoot, and medial and lateral heel	Bluetooth	Yes (no details)	–	–	–	–	–
Rajala et al., 2017 [40]	Insole	Plantar pressure	Piezoelectric sensor (PVDF)	8	Hallux, two on the 1st metatarsal head, 2nd, 3rd, 4th and 5th metatarsal head and heel	Wired	–	–	Author presents measurements with patients up to ≈ 500 kPa	28.5 ± 1.0 pC.N^−1^	10–40 °C	–
Raviglione et al., 2017 [73]	Wearable system	Plantar pressure	Piezoresistive textile sensor	1	Plantar region (to be placed on the area of interest)	Bluetooth	–	–	0–845.5 kPa	–	–	–
Suresh et al., 2014 [70]	Footwear-coupled plantar system	Plantar pressure	Fiber optic sensor (FBG)	2	Forefoot and heel	Wired	–	200 Hz	Author presents measurements with patients up to ≈ 700 kPa	1.3 pm.kPa^−1^ 0.8 kPa	–	–
Suresh et al., 2015 [38]	Insole	Plantar pressure	Fiber optic sensor (FBG)	4	1st and 5th metatarsal head, medial and lateral heel	Wired	–	–	Author presents measurements with patients up to ≈ 1400 kPa	1.2 pm.kPa^−1^ ≈0.8 kPa	–	–
Takano et al., 2014 [48]	Hybrid footwear-coupled plantar system (combination of F-Scan insole and uniaxial shear sensors)	Plantar pressure and shear force	F-Scan: resistive; Uniaxial shear sensor: Magnetic variation (Keisoku Support Ltd., Hiroshima)	F-Scan: 954 (variable with insole number); Uniaxial: 2	F-Scan: the entire plantar region; Uniaxial: 1st and 2nd metatarsal head	Wired	–	F-Scan: 50 Hz; Sensors: 500 Hz	F-Scan: up to 862 kPa; Sensors: up to 200 N	F-Scan: 3.9 sensors per cm^2^ (spatial resolution) Sensors: 6.1 × 10^−4^ kgf	–	F-Scan: 5 a 15; Sensors: no details
Tan et al., 2021 [45]	Insole	Plantar pressure	Piezoresistive textile sensor	6	Hallux, 1st and 4th metatarsal head, lateral midfoot, and medial and lateral heel	Bluetooth	Yes (no details)	–	Up to 800 kPa (0–100 kPa stability guarantee)	3.96 kPa^−1^ in the range 0–36 kPa (0.49 kPa^−1^ for upper range)	–	1000
Tang et al., 2023 [52]	Insole	Plantar pressure and shear force	Capacitive sensor	4	Hallux, 1st and 5th metatarsal heads and heel	Wireless (no details)	Yes (no details)	100 Hz	–	–	–	–
Wang et al., 2005 [50]	Sensor	Plantar pressure and shear force	Fiber optic (no detail)	Not applicable	Not applicable	Not applicable	Not applicable	Not applicable	–	Detection limit for shear: 2.2 N	–	–
Wang et al., 2008 [68]	Sensor	Plantar pressure	Fiber optic (PMDS)	Not applicable	Not applicable	Not applicable	Not applicable	Not applicable	Up to 500 kPa	0,1% 0.027 N	–	–
Wang et al., 2020a [37]	Insole (control system integrated in the sole of the shoe)	Plantar pressure	Flexible piezoresistive sensor	8	Hallux, 1st, 2nd/3rd and 4th/5th metatarsal heads, medial and lateral midfoot, medial and lateral heel	Bluetooth	3.7 V, 450 mAh; up to 20 h	20 Hz	0–45 N or 0–600 kPa	0.0105 N^−1^	–	Up to 10^5^
Wang et al., 2020b [49]	Sensor	Plantar pressure and shear force	Triaxial inductive sensor	Not applicable	Not applicable	Not applicable	Not applicable	Not applicable	Shear (x, y): −160 to 160 kPa; Vertical pressure (z): 0–1047 kPa	x: 489.1 nH mm^−1^ y: 494.6 nH mm^−1^ z: 187.1–1210 nH mm^−1^	–	–
Wertsch et al., 1995 [53]	Plantar system for attaching to the shoe	Plantar pressure	Resistive force sensor (FSR, Interlink Electronics)	8	Hallux, 1st, 2nd, 4th and 5th metatarsal heads, medial and lateral midfoot and heel	Wired	–	35 Hz	–	–	–	–
Zhu et al., 1991 [43]	Insole	Plantar pressure	Resistive force sensor (based on conductive polymer pressure sensor; FSR Interlink Electronics)	7	Four on the metatarsal heads, hallux and posterior and anterior heel	Wired (system in a backpack on the user's back)	Yes (no details)	20 Hz	Author presents measurements with patients up to 657 kPa	–	Sensors calibrated at 36 °C	–
Zhu et al., 1993 [80]	Insole	Plantar pressure	Resistive force sensor (based on conductive polymer pressure sensor; FSR, Interlink Electronics)	7	Four on the metatarsal heads, hallux and posterior and anterior heel	Wired (system in a backpack on the user's back)	Yes (no details)	35 Hz	0–1.2 MPa	0.3–30 mV kPa^−1^	Sensors calibrated at 36 °C	–

The assessment of data quality from the studies was not conducted due to several reasons. Firstly, the great diversity among the identified studies, coupled with the lack of a universal methodology and specific guidelines (particularly in studies at the intersection of engineering and health disciplines), made it challenging to establish a standardized approach. Additionally, some studies lacked a defined target sample and objective classification of the study type. This diversity not only prevented the assessment of study quality but also rendered the conduct of a meta-analysis unfeasible.

### 3.1 What is the typology of the devices and what can they measure?

***Systems available on the market.*** The market offers some products intended for both users and healthcare professionals, all aimed at measuring and evaluating plantar pressures. These products primarily consist of systems intended to be inserted inside shoes, typically in the form of insoles or socks (although socks are less common compared to insoles). Despite their shared purpose of measuring plantar pressures, these systems vary in their technological characteristics [30]. Table 1 provides a comparative summary of the technology used by each system and their distinctive features, with complementary information taken from manufacturers' websites.

Among these systems, the F-Scan systems [31] and Pedar insole [32], both in the insole format for insertion into shoes, stand out as the most widely used for assessing plantar pressures, 44.7% and 37.2% respectively. Of all the devices available, the Pedar insole system is considered the gold standard and one of the most established insole-based plantar pressure measurement systems on the market [26].

Many of the devices can pair and communicate with smartphones through mobile applications to register the data collected in the measurements and access other features. These applications enhance user interaction by presenting data in the form of graphs and colorful mappings indicating areas of plantar pressure, while also issuing alerts for potential occurrences of high pressures. However, it's worth noting that most of these applications are primarily aimed at the sports market, focusing on data collection for athlete performance analysis rather than on preventing high pressure injuries in diabetic foot conditions. Nonetheless, there are also some applications available in this field [22,26,30,33].

***Systems and sensors under development.*** Although some equipment is already available on the market for measuring pressure inside the shoe, the development of new technologies is essential. Solutions with new sensors, wearables and telehealth approaches capable of remotely monitoring a wider set of variables representing the main risk factors of patients with foot pathology are increasingly sought [34].

The evolution of developed equipment shows that the concept of an insole to assess plantar pressures is predominant [[35], [36], [37], [38], [39], [40], [41], [42], [43], [44], [45]]. While systems for measure plantar pressures are prevalent, it's noteworthy that some authors advocate for a hybrid model, simultaneously measuring shear forces in the horizontal plane along with vertical pressures on the shoe insole [35,36,[46], [47], [48], [49], [50], [51], [52]]. Shear results from friction of the foot with the insole itself, which may indicate potential overheating of the skin and an increased risk of injury [35,36].

These devices require a control unit to process the acquired data. In recent developments, proposals for system concepts that are fully integrated into the structure of the shoe have emerged. The entire technology, especially its control unit, is now embedded in the sole of the shoe, giving the appearance of a conventional shoe [37]. For a more comprehensive analysis, Table 2 presents a comparative summary of the characteristics of the systems under development.

### 3.2 What technology is used?

**Fig. 3:**
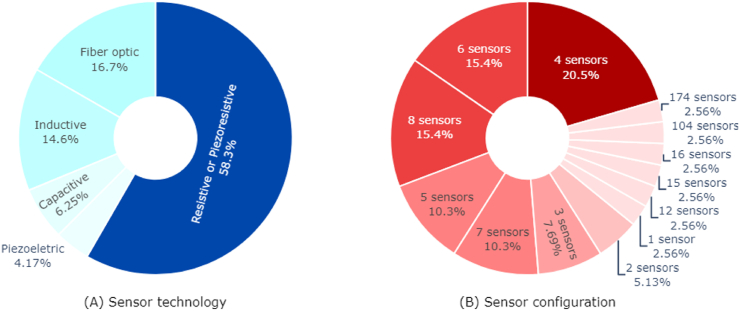
Predominant sensor technologies (A) and distribution of the most commonly used sensor configurations (B).

This review identified several types of technology integrated into the proposed measurement systems, including resistive technology [44,[53], [54], [55], [56], [57], [58], [59], [60], [61], [62], [63], [64], [65]], piezoelectric [39,40,46], capacitive [41,52,66], inductive [49,51], fiber optics [38,50,[67], [68], [69], [70], [71], [72]] and textile technology with piezoresistive features [[73], [74], [75]]. In Fig. 3A, the graph depicts the most prevalent technology used, along with its associated percentage.

Currently there is a wide variety of sensors to measure the forces and pressures between the foot and the interior of the shoe, including those used for monitoring the human gait. The most used sensors that can be incorporated into pressure measuring devices in footwear are classified into the following categories based on their operating principle:

a) resistive sensors: this is the most widely used type of sensor in various applications. They consist of an active area filled with a conductive polymer or ink with conductive properties. When pressure is applied to this area, the material's resistance decreases with increasing pressure. The behavior of these sensors is well known and exhibits an almost linear and predictable response within a certain range of pressure values and operating temperatures [26,30,[76], [77], [78]]. The resistive sensors used are mostly force-sensing resistors (FSRs) [61,62], although other applications use Velostat, a polymeric film that follows the same behaviour as this type of sensor [[63], [64], [65]];
b) piezoresistive sensors: like resistive sensors, the entire body of these sensor is typically made of piezoresistive material. They are designed to react when the material is stretched, resulting in a variation in electrical resistance. This resistance is measured and converted into a corresponding pressure value [26,30,77];
c) piezoelectric sensors: these sensors utilize the piezoelectric effect, generating an electrical voltage from applied mechanical pressure. They convert mechanical action into an electrical charge, which is measured and converted into a pressure value The active area can be produced using polyvinylidene difluoride (PVDF), a type of thermoplastic fluoropolymer [26,30,77,78];
d) capacitive sensors: Comprising two conductive plates with an insulating material between them, capacitive sensors exhibit a decrease in distance between the plates when pressure is applied. This compression of the insulating material leads to a variation in sensor capacitance and consequently its electrical voltage, representing a pressure value [26,30,77,78];
e) inductive sensors: these sensors generate a variable electromagnetic field through a coil. When a metallic material enters this field, eddy currents are induced, altering the coil's inductance value. This change corresponds to the observed deformation resulting from applied pressure [49];
f) textile sensors: utilizing conductive inks combined with textile material or integrated conductive wires within the textile matrix. A textile matrix can incorporate a large number of sensors. However, they may exhibit non-linearity and sometimes a significant hysteresis [26,30];
g) fiber optic sensors: typically produced in a polymer (POF, Polymer Optical Fiber; PMDS, Polydimethylsiloxane), fiber optic sensors consist of a light emitter and a light receiver with a specific wavelength. When the fiber is pressed, the Fiber Bragg Gratings (FBG) sensor measures deformation by varying the photo-elastic constant of the fiber and a change in microstructure period, resulting in a change in Bragg wavelength [38,50,[67], [68], [69], [70], [71], [72]].

### 3.3 What is the configuration and number of sensors?

Typically, pressure sensors for measuring plantar pressures on a patient's foot are positioned on the inner base of the shoe, either incorporated in the insole itself or within a secondary removable insole [77]. These sensors can be employed individually to obtain discrete measurements of specific region of clinical interest or arranged in an array for higher spatial resolution and sensor density. In the latter configuration, greater resolution is achieved by distributing sensors across the intended skin region for evaluation [79]. These sensors are usually placed at strategic points, which are well-defined in the literature as areas where high pressures commonly occur, considering various foot types such as normal, planus and cavus foot [2]. Some authors propose systems with one until eight measurement points in the plantar region [37,54,55]. However, configurations with four (20.5%), six (15.4%) or eight (15.4%) measurement points/sensors are more prevalent [37,[39], [40], [41],^43^,^58^], as they cover the key points considered clinically relevant for assess plantar high pressures [2]. In Fig. 3B, the graph illustrates the most used sensor configuration along with the corresponding percentage.

The critical points where sensors are typically positioned include the plantar region of the hallux, the heads of the first, second/third and fourth/fifth metatarsals, the medial and lateral midfoot, and the medial and lateral heel [37,54,55]. Some commercially available devices offer more comprehensive foot mapping, such as the F-Scan insole, which can feature a spatial distribution of approximately 960 sensors [31].

The measurement area covered by each sensor in a given configuration and arrangement in the plantar region depends on the sensor density and desired spatial resolution. In the most common model, with FSR sensors (31.1%) distributed at discrete points across the plantar region, the standard diameter of the sensor's active area is approximately 15 mm [42,43,[53], [54], [55],^58^,^59^,^80^]. However, in other configurations, authors choose for smaller diameters, ranging from 4 to 10 mm, considering different technologies used and systems [[35], [36], [37],^44^,[46], [47], [48],^56^,^57^,^81^]. There are other configurations with greater spatial resolution of sensors, achieved through a matrix produced with Velostat film, which can reach a hundred square sensors measuring 5 mm on each side [[63], [64], [65]].

### 3.4 What are the operating conditions?

All systems require an additional external unit for acquisition, processing, and power supply, which is sometimes included in the external unit's box. Most systems, as reported by Refs. [41,46,47,55,81,82], have an acquisition rate around 100 Hz. This external unit is typically attached to the patient's leg or waist and connected with wires to the measuring device inside the shoe [43,46,54,56,80].

Some authors propose systems with a wireless communication-enabled control unit to enhance patient mobility and utility outside of the clinical environment. For example, systems with Bluetooth or Wi-Fi connectivity [37,39,41,45,57,73,74], powering by battery, but with varying levels of autonomy, ranging from a few hours to several days, depending on usage cycles [39,41,54,69,83].

These devices are subject to operating conditions imposed by the individual characteristics of each user's foot. The upper limit of measurement can vary from approximately 500 kPa to 1.4 MPa, as reported by Refs. [37,45,49,55,68,73,75,80]. Information regarding the sensitivity, resolution of measurement amplitude, tolerated temperatures, humidity limits and number of usage cycles is scarce. Some authors only guarantee sensor stability for about 1000 usage cycles [45] or up to 100,000 cycles [37] for singular sensors placed in specific insole locations. In other cases, they guarantee stability for only five to 15 usage cycles with intense activity, such as the F-Scan insoles [31], sometimes combined in a hybrid model with other sensors [35,36,47,48].

### 3.5 What results do these technologies offer?

When a patient uses a certain device, either on their own initiative or on the advice of the attending physician, the goal is to objectively measure the plantar pressures involved between the foot and the shoe used. Disparities in plantar pressure values are found in the literature, which are intrinsically linked to factors such as the type of footwear used (model, material, lacing method), the chosen measurement system, the method of device insertion and accommodation within the footwear and the characteristic anatomy of each patient [44,[84], [85], [86], [87]]. Table 3 demonstrates the average plantar pressure measurements obtained in studies conducted with diabetic and non-diabetic (healthy) volunteer participants.

**Table 3:** Average values (rounded to the unit) of plantar pressure measured in different studies with diabetic and non-diabetic (healthy) participants. Abbreviations: MH 1 - first metatarsal head, MH 2/3 - second/third metatarsal heads, MH 4/5 - fourth/fifth metatarsal heads, ML - midfoot lateral.

Author, year	Target Sample	Used device	Average plantar pressure values (kPa)					
			Hallux	MH 1	MH 2/3	MH 4/5	ML	Heel
Amemyia et al., 2016 [46]	3 non-diabetics	Plantar system with triaxial pressure sensors (system developed by the author)	–	172	319	158	–	–
Ledoux et al., 2013 [127]	596 diabetics with: ulceration potential & with ulcer	F-Scan [31]	200 & 172	242 & 383	308 & 362	177 & 220	141 & 267	266 & 241
Lung et al., 2016 [94]	8 non-diabetics & 19 diabetics	F-Scan [31]	297 & 489	320 & 510	400 & 390	– & –	– & –	280 & 280
Martinez et al., 2008 [85]	45 non-diabetics	BioFoot [163]	178	359	585	159	–	–
Ostadabbas et al., 2012 [44]	11 non-diabetics	Insole with FSR sensors (system developed by the author)	124	95	114	68	–	185
Pataky et al., 2005 [60]	15 non-diabetics & 15 diabetics (right foot)	Insole with FSR sensors (system developed by the author)	101 & 205	160 & 137	221 & 205	97 & 160	– & –	321 & 187
Pataky et al., 2005 [60]	15 non-diabetics & 15 diabetics (left foot)	Insole with FSR sensors (system developed by the author)	104 & 165	146 & 140	220 & 179	91 & 174	– & –	298 & 184
Reints et al., 2017 [136]	30 non-diabetics	Pedar insole [32]	139	98	108	91	25	130

Each patient has a pressure threshold, above which they may be at risk. With the help of these measurement technologies, it is possible to determine the generally advised maximum thresholds over time. According to sources [2,88,89], reducing plantar pressure to values below 200 kPa may be crucial for favorable ulcer treatment and prevention of ulcer recurrence. A reduction ≥30% in peak pressure during walking (compared to current therapeutic footwear for patients with diabetes) is advised to ensure relief of plantar pressure at high pressure sites [2,88,89]. Thus, evaluating plantar pressure values below 200 kPa (200 kPa ≅ 2 kgf. cm^−2^) with a validated and properly calibrated system with a measurement sensor with an active area of 2 cm^2^ is recommended [2].

These systems are widely used in sports and clinical settings to assess human gait and foot-related disorders. Analyzing plantar high pressures can provide important insights into various clinically relevant parameters, including plantar pressure at discrete points, whole foot average pressure, critical peak pressures, center of pressure, displacement and velocity metrics, balance and human gait [30]. The results obtained are fundamental not only for detecting gait and foot-related abnormalities but also for monitoring various pathologies, such as the risk of ulceration from loss of sensation in the feet resulting from diabetic neuropathy [22,25,30].

**Fig. 4:**
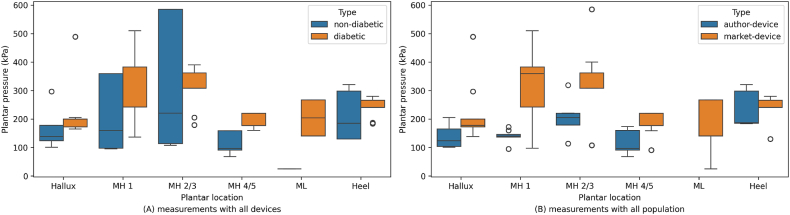
Distribution of average plantar pressures between the non-diabetic and diabetic population through measurements with all devices (A) and between the group of author and market available devices with measurements from all participants (B). The studies do not present sufficient data for ML plantar location to compare author-devices vs. market-devices. Abbreviations: MH 1 - first metatarsal head, MH 2/3 - second/third metatarsal heads, MH 4/5 - fourth/fifth metatarsal heads, ML - midfoot lateral.

**Fig. 5:**
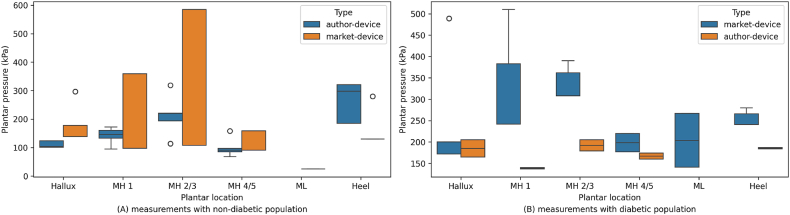
Distribution of average plantar pressures of the non-diabetic population (A) and the diabetic population (B) both through measurements with author devices and those available on the market. The studies do not present sufficient data for ML plantar location to compare author-devices vs. market-devices. Abbreviations: MH 1 - first metatarsal head, MH 2/3 - second/third metatarsal heads, MH 4/5 - fourth/fifth metatarsal heads, ML - midfoot lateral.

Following the utilization of these measurement technologies to assess plantar pressures and their clinical implications, it becomes essential to visually examine the distribution of average plantar pressure data among different patient groups and device types. Fig. 4, Fig. 5 present detailed representations of the average plantar pressure measurements, derived directly from the data presented in Table 3. These data were extracted from studies conducted by respective authors without additional processing, only a classification by groups was made and the sample size of each study was considered for subsequent statistical analysis. Fig. 4A shows the distribution of average plantar pressures between the non-diabetic and diabetic population through measurements with all devices. The data represented in Fig. 4B are between groups of author-devices and market-devices with measurements from the entire participant population. Fig. 5A shows the distribution of average plantar pressures for the non-diabetic population, and in Fig. 5B for the diabetic population, both through measurements with author-devices and those available on the market. These graphical depictions serve as important tools for further analysis and interpretation of the obtained data, clarifying possible disparities and trends in plantar pressure distribution across various conditions and measurement modalities. Additionally, Table 4 presents the results of the Mann-Whitney test for comparing plantar pressures between non-diabetic and diabetic patient groups, as well as between market-devices and author-devices based on plantar pressure measurement locations. Some variables are not reported due to missing data, as not all authors measure pressures with sensors in the same positions. A significance level (*α*) of 0.01 was applied and results with a *P* value < 0.01 indicate a statistically significant difference between the comparison groups.

**Table 4:** Results of the Mann-Whitney test (*α* = 0.01) for comparison of plantar pressures between non-diabetic and diabetic patient groups, as well as between market-devices and author-devices. Abbreviations: MH 1 - first metatarsal head, MH 2/3 - second/third metatarsal heads, MH 4/5 - fourth/fifth metatarsal heads, ML - midfoot lateral, NA - not applicable values due to lack of data to compare. Only underlined *P* values do not represent statistically significant differences in plantar pressures between groups.

Mann-Whitney test	*P* value					
	Hallux	MH 1	MH 2/3	MH 4/5	ML	Heel
non-diabetic vs. diabetic (all devices)	<0.0001	<0.0001	0.0081	<0.0001	<0.0001	0.5504
market-device vs. author-device (all population)	<0.0001	<0.0001	<0.0001	<0.0001	NA	0.0457
market-device vs. author-device (non-diabetic population)	<0.0001	<0.0001	0.0082	<0.0001	NA	<0.0001
market-device vs. author-device (diabetic population)	0.8679	<0.0001	<0.0001	<0.0001	NA	<0.0001

## 4 Discussion

The aim of this systematic review was to identify devices for measuring plantar pressure exclusively inserted, or able to be inserted and removed, within footwear suitable for patients with diabetic foot. Of the devices identified and available on the market, instrumented insoles for insertion into footwear are dominant. The same is true for new technologies under development, with insoles being the most widely studied and used concept.

Most of the devices have the advantage of enabling wireless data communication and possibility of pairing with a smartphone via an app. They make it possible to acquire values in real time while the user is performing his normal movements. It also gives the user the possibility to view some instant results of interest and receive alerts in case of plantar high pressure [37,39,73,74,90,91]. On the other hand, a disadvantage common to almost all devices are that their control unit and data storage is usually fixed on the leg or waist of the user, which can become a nuisance and even induce small changes in human gait [43,46,54,56,80]. Recent developments are exploring new approaches to fully integrate the control unit into the sole of the shoe, aiming to provide a more natural user experience [37].

Two factors to consider, given that this equipment is intended for daily use rather than solely in a clinical environment, are battery autonomy and the number of allowed usage cycles to ensure data quality. Some systems [31,32] claim to have features for high-performance activities, such as sports or more dynamic human gait, however, in the case of commercial F-Scan insoles, their durability does not go beyond 15 uses [31]. As such, in other systems, the durability of the sensors should also be considered, because due to the flexion of the shoe during walking and the friction of the foot itself, they will be subject to a high wear [79].

Another additional factor to consider is the insertion of any plantar pressure measuring system inside the shoe. The available space of the shoe must be balanced with the space taken up by the device [79]. As referred to by the international guidelines [2] on the assessment of the diabetic foot and recommendations on the appropriate footwear to be worn, consideration must be given to the internal structure of the footwear or any foreign body that may add relief. Due to the patient losing protective skin sensitivity and pain sensation being compromised [2], anybody that adds relief inside the shoe can lead to trauma, thus becoming a problem in these cases [79]. For this reason, sensors incorporated within the insole itself or under the insole are the most effective solution [37]. The insertion of the equipment inside the shoe must not increase the risk of injury, and the presence of the device must be as unobtrusive as possible to avoid inducing forced changes in human gait [92,111,112,114,116,117,[129], [130], [131],^134^,^137^,^138^,^142^,^148^,^149^].

The most established technologies for integrating these systems, which have been used for years, are resistive sensors. For their simplicity and knowledge of their behavior, these sensors are commonly selected for instrumented removable insole solutions or systems integrated into the shoe [42,44,[53], [54], [55], [56], [57], [58], [59], [60]]. Other technologies such as fiber optic sensors, used in more recent approaches, are beginning to show some results, but still have some difficulties in sensor calibration and characterization. The control unit, which needs a light emission source, is also still a constraint because of its dimensions [50]. New inductive sensors in plantar systems are being developed, and besides measuring plantar pressure, they also measure the shear forces inherent to the friction between the foot and the inside of the shoe. This concern in evaluating the shear forces is pertinent because through friction there is an increase in the temperature of the cutaneous region and it may trigger an ulcer [49,51].

The approach with discrete sensors is the simplest, even from the point of view of control electronics. The configuration with eight sensors located in the hallux, metatarsal heads, midfoot and heel proves to be the most pragmatic decision because it covers the crucial points where high pressures occur [37,40,41,53,74]. The advantage of this type of configuration over a sensor array with higher spatial resolution is the high sampling frequency in signal acquisition (typically 100–200 Hz or higher) since the number of sensors used is smaller and makes possible a faster reading of the entire measurement system [35,41,44,46,47,55,70,81,82].

Although several studies fail to adequately communicate the characteristics of the product and it is not possible to effectively compare their capabilities, the results obtained using these technologies still show that it is possible to measure and evaluate plantar pressures. However, average values of plantar pressure, like those presented in Table 3, may depend on the anatomical characteristics of the foot of each patient, the type of footwear and other conditions. Nevertheless, the plantar pressure peaks may occur in a wider range of values. For example, some authors [44,[85], [86], [87]] recorded values from 100 kPa up to 500 kPa for the hallux region, up to approximately 700 kPa for the first metatarsal head and values exceeding 950 kPa for the second metatarsal head [44,[85], [86], [87]]. There seems to be a slight tendency for the group of people with diabetes with greater susceptibility to ulceration in a certain plantar region to be those who have higher pressure values compared to healthy people. Even so, there are healthy people with high pressure values, for example, in the heel [94]. This reveals the characteristics of human gait that vary from person to person, which requires adapting a certain pressure threshold case-by-case basis as a strategy to avoid ulcerations [95].

After analyzing Table 4, which presents the results of the Mann-Whitney test comparing plantar pressures between groups of people with diabetes and non-diabetic, as well as between market-devices and author-devices based on the locations of plantar pressure measurements, it is observed that there are statistically significant differences in practically all locations, except for two locations in three different comparison tests. Specifically for the heel, in comparisons between non-diabetic patients and diabetics with all devices, and when comparing market-devices vs. author-devices across the entire population, *P* values of 0.5504 and 0.0457 were obtained, respectively. These suggest the absence of a statistically significant difference between these groups at this location. Similarly, when comparing market-devices vs. author-devices for the diabetic population, no statistically significant difference was found for the hallux, with a *P* value of 0.8679. However, the most *P* values (<0.0001) suggest a significant difference in certain measurement locations between the groups evaluated. It is important to highlight that some variables do not present values due to lack of data, as not all authors measure pressures with sensors in the same positions.

Although we made efforts to produce a rigorous systematic review, it is important to recognize some limitations that may have impacted our analysis. The diversity of studies included in the review may have influenced the consistency of our approach and the interpretation of results, due to variations in the methodology each used, target sample and classification of studies. This diversity also made it impossible to assess the quality of studies, including carrying out a meta-analysis, due to the lack of a universal methodology with specific guidelines for this type of studies. Although with a smaller impact, it is important to mention some aspects that can be considered as limitations during the research and article selection process. Screening restricted to English only, document and source type may have resulted in the loss of some relevant studies. Although we explored four databases (some multidisciplinary) recognized by the scientific community, it is possible that other sources of literature were not considered. These considerations highlight the necessary prudence in interpreting results and suggest that, particularly for studies using plantar pressure measurement technologies, a universal methodology with its own guidelines is necessary.

## 5 Conclusion

This systematic review highlighted the abundance of equipment available on the market and the ongoing trend of new developments. It has also revealed insufficient technical data, making it difficult to make a comprehensive comparison of device and sensor characteristics, particularly regarding operating conditions, sensitivity, resolution, temperature and humidity tolerance and the number of admissible usage cycles.

Tactically placed sensors at points associated with high-risk areas of peak plantar pressure are widely used, enabling the calculation of peak or average plantar pressures in these regions. The frequent use of plantar pressure analysis to customize insoles and footwear tailored to individual patient needs may effectively relieve plantar pressure in patients with diabetic foot. Resistive sensor technology emerges as the most practical choice for integration into devices due to its widespread use and ease of implementation. The use of these technologies with the different techniques is recommended to try to assess and predict risk situations.

The analysis of average plantar pressure values, comparing patients with diabetes and potential complications with healthy individuals, suggests a trend in which it is possible to differentiate the two groups by plantar pressure level in some areas of the foot. This statement may be partially supported due to the significant differences obtained in the comparison tests. Recognizing the limitations of the studies' data sample, it is important to highlight that statistically significant differences between different types of devices are also suggested. This can be a critical factor and highlights the importance of individualized patient assessment using consistent measuring devices. By evaluating each patient with the same type of device, clinicians may be able to minimize measurement error and obtain more objective data to characterize plantar pressure patterns and assess the progression of injury risk over time.

Although it was not the focus of this review, the synthesis of the information collected allows considering that the approaches used to assess the shear forces are relevant [49,51], since the friction of the foot inside the shoe during gait is extremely important due to the overheating of the skin and the imminent risk of injury [133]. Another aspect excluded from the focus of this review, but currently with increasing importance, refers to the patient's adherence to these technologies for daily use when prescribed by your attending physician. Poor adherence appears to be common in offloading strategies for a variety of reasons.

## Protocol and registration

The protocol of this systematic review was not registered.

## Ethics approval

Not applicable within the scope of this systematic review.

## Funding sources

This work was developed under a PhD grant (DOI: 10.54499/UI/BD/151285/2021) awarded to PCM and funded by the Portuguese Foundation for Science and Technology (10.13039/501100001871FCT, Portugal). It also had the support of the “Smart-Health-4-All – Smart medical technologies for better health and care” project (POCI-01-0247-FEDER-046115; LISBOA-01-0247-FEDER-046115), which was co-financed by Portugal 2020, under the Operational Program for Competitiveness and Internationalization (10.13039/501100011929COMPETE 2020) through the European Regional Development Fund (10.13039/501100008530ERDF).

## Data availability statement

This work is a systematic literature review based on searches in the previously mentioned electronic databases, therefore, the data supporting this study were not stored in any other repository. All additional data and information are available upon request from the corresponding author.

## CRediT authorship contribution statement

**Pedro Castro-Martins:** Writing – review & editing, Writing – original draft, Visualization, Validation, Methodology, Investigation, Formal analysis, Data curation, Conceptualization. **Arcelina Marques:** Writing – review & editing, Writing – original draft, Validation, Supervision, Methodology, Data curation, Conceptualization, Funding acquisition. **Luís Coelho:** Writing – review & editing, Writing – original draft, Visualization, Validation, Supervision, Methodology, Investigation, Funding acquisition, Formal analysis, Data curation, Conceptualization. **Mário Vaz:** Writing – original draft, Validation, Supervision, Funding acquisition, Conceptualization. **João Santos Baptista:** Writing – original draft, Visualization, Validation, Supervision, Project administration, Conceptualization.

## Declaration of competing interest

The authors declare that they have no known competing financial interests or personal relationships that could have appeared to influence the work reported in this paper.
